# A comparative study on the frequency of simulation-based training and assessment of non-technical skills in the Norwegian ground ambulance services and helicopter emergency medical services

**DOI:** 10.1186/s12913-018-3325-1

**Published:** 2018-07-03

**Authors:** Henrik Langdalen, Eirik B. Abrahamsen, Stephen J. M. Sollid, Leif Inge K. Sørskår, Håkon B. Abrahamsen

**Affiliations:** 1 Department of Safety, Economics and Planning, University of Stavanger, Faculty of Science and Technology, Stavanger, Norway; 2 Department of Quality and Health Technology, University of Stavanger, Faculty of Health Sciences, Stavanger, Norway; 3 Prehospital Division, Stavanger University Hospital, Stavanger, Norway; 4 Department of Anaesthesiology and Intensive Care, Stavanger University Hospital, Stavanger, Norway

**Keywords:** Non-technical skills, Helicopter, Ambulance, Emergency medical service, Simulation-based training, Assessment, Learning

## Abstract

**Background:**

Inadequate non-technical skills (NTSs) among employees in the Norwegian prehospital emergency medical services (EMSs) are a risk for patient and operational safety. Simulation-based training and assessment is promising with respect to improving NTSs. The frequency of simulation-based training in and assessment of NTSs among crewmembers in the Norwegian helicopter emergency medical service (HEMS) has gained increased attention over recent years, whereas there has been much less focus on the Norwegian ground emergency medical service (GEMS). The aim of the study was to compare and document the frequencies of simulation-based training in and assessment of seven NTSs between the Norwegian HEMS and GEMS, conditional on workplace and occupation.

**Method:**

A comparative study of the results from cross-sectional questionnaires responded to by employees in the Norwegian prehospital EMSs in 2016 regarding training in and assessment of NTSs during 2015, with a focus on the Norwegian GEMS and HEMS. Professional groups of interest are: pilots, HEMS crew members (HCMs), physicians, paramedics, emergency medical technicians (EMTs), EMT apprentices, nurses and nurses with an EMT licence.

**Results:**

The frequency of simulation-based training in and assessment of seven generic NTSs was statistically significantly greater for HEMS than for GEMS during 2015. Compared with pilots and HCMs, other health care providers in GEMS and HEMS undergo statistically significantly less frequent simulation-based training in and assessment of NTSs. Physicians working in the HEMS appear to be undergoing training and assessment more frequently than the rest of the health trust employees. The study indicates a tendency for lesser focus on the assessment of NTSs compared to simulation-based training.

**Conclusion:**

HEMS has become superior to GEMS, in terms of frequency of training in and assessment of NTSs. The low frequency of training in and assessment of NTSs in GEMS suggests that there is a great potential to learn from HEMS and to strengthen the focus on NTSs. Increased frequency of assessment of NTSs in both HEMS and GEMS is called for.

## Background

The Norwegian prehospital emergency medical service (EMS) is an integrated part of the Norwegian preparedness system [1], dedicated to providing immediate medical attention and delivery of care outside the hospitals to the Norwegian population in the case of emergency, acute illness or critical injury [2, 3]. The helicopter emergency medical service (HEMS) and ground emergency medical service (GEMS), i.e. ambulance cars and boats, constitutes the major part of the Norwegian EMS. In Norway, commercial flight operators run the HEMS operations on behalf of the regional health trusts, whereas the regional health trusts are responsible for the EMSs in their local region. Objectives and tasks within HEMS and GEMS are similar. However, team composition, education, medical care and treatment processes (e.g. a greater number of advanced medical interventions in HEMS [4, 5]), as well as the physical environments, differ substantially.

In both prehospital EMSs, educated, knowledgeable and skilled personnel are required to appraise the situation and adopt the appropriate approach in a vast diversity of encountered circumstances [6, 7]. Intense time pressure, complex problems, uncertainties, high stakes, in addition to a number of individual challenging and interactive tasks of medical, technical and multidisciplinary character, are, among others, common denominators for EMS personnel [8]. The complexity of the prehospital EMS makes the operating environment prone to human error [6, 9, 10].

Human factors pertain to nearly all aspects of the EMSs [11]; thus, preventing human error is paramount [7, 12]. Poor clinical judgments by EMS personnel can reduce patient safety [13]. Efficient situation awareness is critical in the EMS domain, as routine behaviours are interspersed with adverse events that may require a higher level of attention [14]. Multidisciplinary crews require good teamwork to ensure the safety of operations and patients [10, 15, 16]. The fact that factors beyond technical skills and knowledge can cause accidents has promoted the transfer of safety management strategies developed for the aviation industry, such as crew resource management (CRM) [17, 18], into the medical domain [19, 20].

Specific interventions, e.g. CRM such as simulation-based training, can reduce the risk of human error by enhancing non-technical skills (NTSs) [18, 19, 21–23], ensuring safe and effective task performance [24]. NTSs complement technical “know-how” types of skills [6] and are commonly referred to as “social, cognitive and personal resource skills” [25]. Seven generic categories of NTSs are often mentioned in relation to safety [25]: decision-making, leadership, communication, situation awareness, teamwork, managing stress, and coping with fatigue.

Simulation-based training in NTSs is a central CRM intervention, recommended to improve the safety culture in prehospital domains [10], where professionals from different backgrounds practise on non-routine behaviours and tasks in safe environments [11]. The research on NTSs and simulations in a prehospital setting is sparse [13]. However, experience from other domains is promising [26, 27]. Simulations performed by emergency medicine residents improved leadership, communication, teamwork and situation awareness [28]. Assessment of a simulation increases learning, thus potentially preventing the repetition of incorrect behaviour [29–31].

Despite the benefits documented in the literature, the effect of CRM on an organization’s outcome (i.e. safety) has not been ascertained [27]. The optimum frequency of CRM interventions is also uncertain, but emerging evidence seems to support high frequency retraining [32]. The opportunity cost of CRM interventions is great, as they are resource-absorbing [33], limited by budget constraints and time to practise. Mapping the frequency of training in and assessment of NTSs is a means to identify the need for such interventions, the development of skills and the resource-effectiveness.

To the extent of our knowledge, the level of training in and assessment of NTSs in the Norwegian GEMS has not been reported in the literature. The level of simulation-based training in and assessment of NTSs in the Norwegian HEMS during 2011 has been documented in the literature [34]. The results indicated a significant difference in the frequency of training in and assessment of NTSs between employees working for the flight operator and those in the health trust.

The aim of this study was to document and compare the frequency of training in and assessment of a generic set of basic NTSs within the Norwegian HEMS and GEMS. We hypothesized that the health trust employees, compared to the flight operator employees, lacked simulation-based training in, and assessment of, NTSs. We also hypothesized that physicians working in the HEMS underwent training and assessment more frequently than the other health trust employees. Finally, we asserted that the difference in frequency of training in and assessment of NTSs between the HEMS and the GEMS has increased in recent years.

## Methods

### Setting

The Norwegian GEMS is considered the backbone of the Norwegian EMS [35]. The most common staffing in the Norwegian GEMS is either one paramedic and one emergency medical technician (EMT) or two EMTs [36], at least one of whom is an authorized EMT [3]. Norwegian EMTs (“Ambulansepersonell”) undergo two years of vocational high school, followed by two years of practical on-the-job training, working as an EMT apprentice, to become authorized. The primary responsibilities of the EMT are transportation, primary survey, initiating medical care and triage on-scene. A paramedic needs an EMT licence and a university college degree of 60 to 180 European Credit Transfer and Accumulation System (ECTS) points [36]. In addition to paramedics and EMTs, physicians and nurses with or without additional authorization as an EMT or other speciality training (e.g. anaesthesia) may supplement the Norwegian GEMS staffing. Professional GEMS groups, employed by the Norwegian health trusts, of interest in the present study are paramedics, EMTs, EMT apprentices, nurses with authorizations as an EMT (referred to as EMT nurses within this paper) and nurses without an EMT certificate (referred to as nurses).

In Norway, the physician-manned HEMS supports the GEMS in emergency missions for patient care and retrieval, in addition to inter-hospital transportation of patients [34], especially when the time dimension is critical. A HEMS crew consists of three members, each of whom belongs to a different profession. The helicopter pilot acts as mission leader, primarily focusing on navigation and flight safety. The HEMS crewmember (HCM) performs rescue operations and has a supporting role in respect of the pilot and physician in different phases of the mission. The physician, who is a certified or in-training anaesthesiologist, is responsible for patient care and medical treatment, both on-scene and during transportation. The Norwegian commercial flight operator employs the pilots and HCMs, whereas the physicians are employed by the local health trusts.

### Questionnaire

The basis of this study was a Norwegian Hospital Survey on Patient Safety Culture (HSOPSC) conducted in 2016, which included data regarding the self-reported frequency of training in and assessment of NTSs during 2015 among EMS professions. The primary focus of the present paper originates from two question categories addressing the extent of simulation-based training in, and assessment of, seven generic NTSs during 2015. The questions were: “*How many times during 2015 did you participate in multi-professional prehospital simulation-based training in the following skill?*” and “*How many times during 2015 were the following of your prehospital skills systematically observed and evaluated*?”. In this text, the formative debriefing of systematic observation and evaluation of NTSs is referred to as the assessment of NTSs. The skills referred to are the following NTSs: decision-making, leadership, communication, situation awareness, teamwork, managing stress, and coping with fatigue. Each question item was answered across a four-point Likert scale (0, 1–2, 3–5, > 5).

### Data collection

Data were collected between October and December 2016. The survey was distributed by e-mail, with a link to a web-based questionnaire (SurveyXact), to all prehospital personnel in the Norwegian HEMS and GEMS. Non-responders received up to five reminders before they were excluded from the study. Employees in the Norwegian Search and Rescue (SAR) services and medical airplanes were not invited, thus leading to an exclusion if such occupations were found among the respondents. Questionnaires returned with unknown profession or occupation were also excluded by listwise removal.

### Statistical analysis

To assess possible differences we dichotomized the question items into “some training/assessment” and “no training/assessment”. Similarities, or dissimilarities, between professions or EMSs of interest are visually presented in bar charts as proportions of individuals (in %) within the respective group.

To support the visual comparison of professional groups, the dichotomized items were used in several two-sided Fisher’s exact tests. Results are presented as numbers (ratios) and *p*-values, where a *p*-value less than 0.05 is considered as statistically significant throughout the paper.

Calculated odds ratios (ORs) present the differences within all six professions working in the health trust, with physicians as the reference group. The results are presented as ORs with associated *p*-values. We used the dichotomized items as dependent variables in a series of logistic regressions, with the professional groups as explanatory variables, to obtain the p-values.

We used the freeware R 3.4.2 for calculations and visualizations of all the results presented in the present paper.

### Ethics, consent and participants

This study was conducted on the approval obtained from the Norwegian Social Science Data Services (NSD; project number 45723). The Regional Committee for Medical and Health Research West-Norway (REK West) evaluated this project as “not mandatory to submit” (Ref. number 2015/2249). All the participants received information about the purpose of the study, and written consent to participate was given at the start of the study, stating that no participants could be identified in published material. The digital questionnaires were treated in confidence.

## Results

**Fig. 1:**
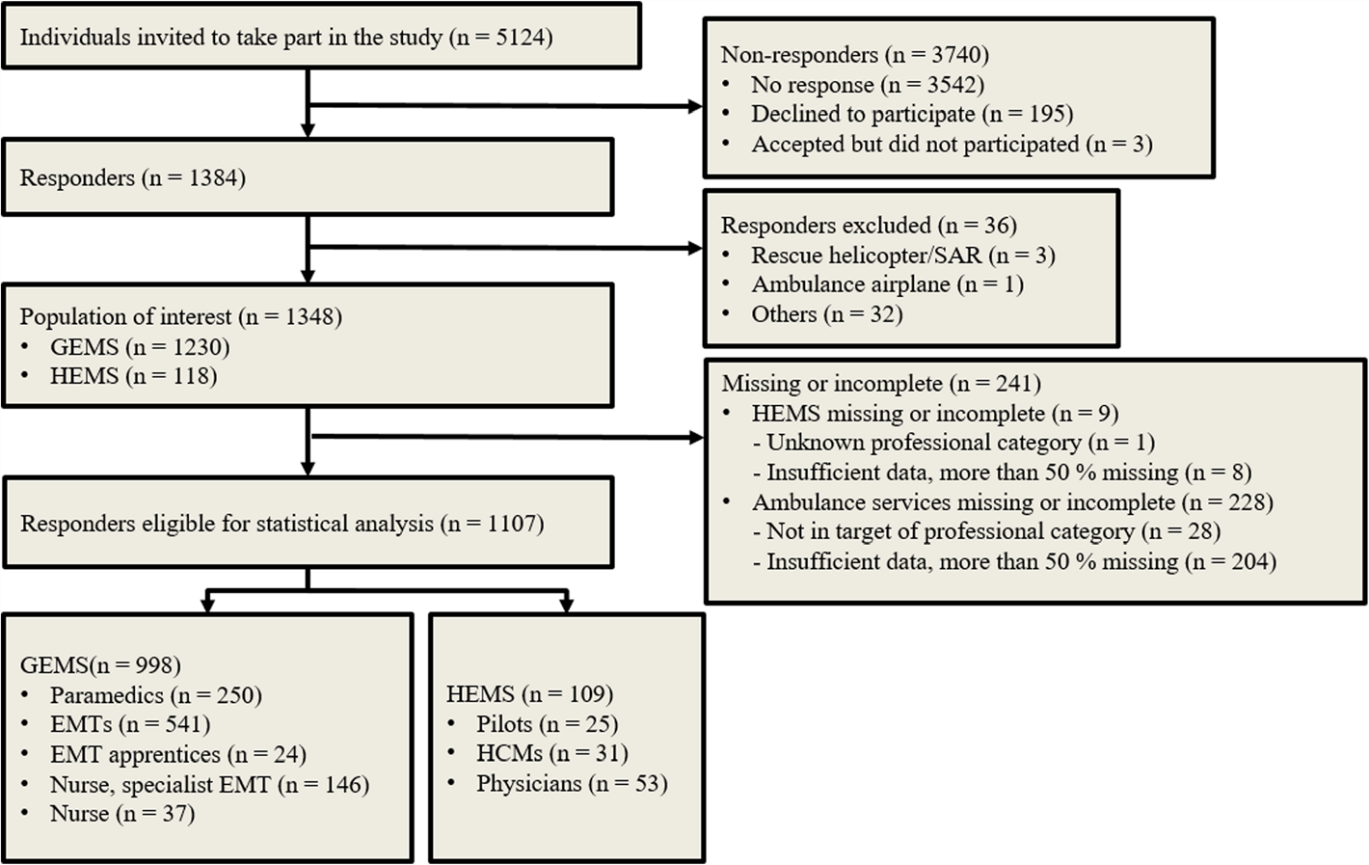
Population map of the participants (number of respondents) in the survey and eligible population used for statistical analysis

The participant flow of respondents who qualified for the statistical analysis is illustrated in Fig. 1. Of the 5124 people invited to participate in the survey, all somehow engaged in the Norwegian EMSs, 4910 and 214 worked for the GEMS and HEMS, respectively. Responders accepting the survey numbered 1384 (response rate of 27.0%). We excluded 36 respondents, as they did not work in an EMS of interest (e.g. employees in SAR services). Among the respondents of interest, 241 respondents were excluded, due to unknown professions, irrelevant professions (e.g. ambulance assistant), and insufficient answers (less than 50%). Of the 1107 responders qualified for statistical analysis, 998 (response rate of 20.3%) worked for the GEMS and 109 (50.9%) for the HEMS. The professional groups of interest are presented in the two lower boxes in Fig. 1.

**Fig. 2:**
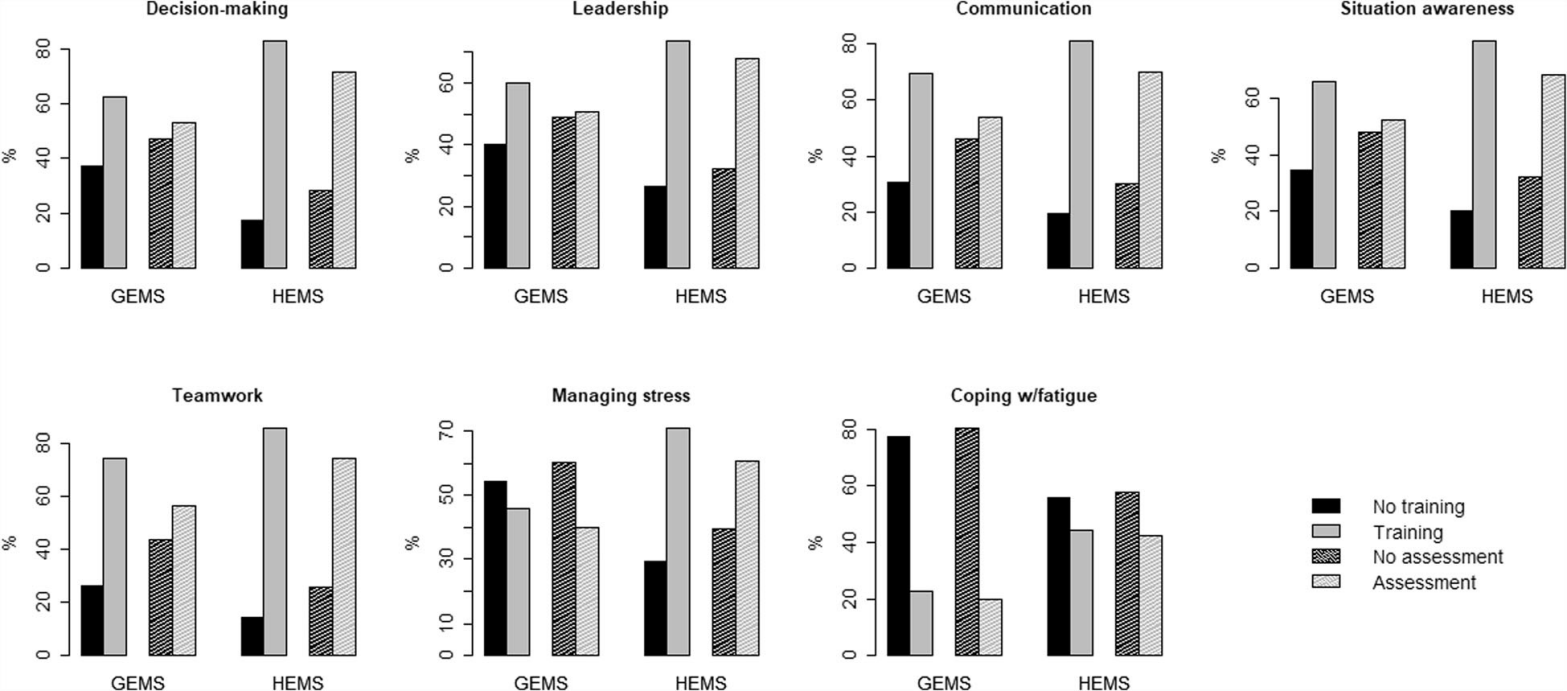
Simulation-based training and assessment (dashed filling) of the seven generic NTSs within the GEMS and HEMS in 2015. All answers from each of the EMSs are dichotomized into no training/assessment or some training/assessment. Proportions of individuals are the relative frequencies (in %) within each EMS

Visual inspection of the frequency of simulation-based training and assessment in the HEMS and GEMS (Fig. 2) indicates that the HEMS personnel are generally exposed to training and assessment of NTSs more frequently than the GEMS personnel. These apparent differences between the two EMSs are all statistically significant, supported by two-sided Fisher’s exact tests (Table 1). In other words, there is a statistically significant association between the amount of training and assessment and the two EMSs. In general, teamwork and coping with fatigue have the highest and lowest frequencies of training and assessment, respectively.

**Table 1:** Numbers (frequencies) of GEMS and HEMS employees undertaking some training in and assessment of the seven NTSs during 2015; Fisher’s exact test proving statistically significant differences

Question category	NTS category	GEMS (*n* = 998)	HEMS (*n* = 109)	*p*-value
	Decision-making	624 (62.5%)	90 (82.6%)	< 0,001
Simulation-based training of NTSs	Leadership	599 (60.0%)	80 (73.4%)	0,007
	Communication	693 (69.4%)	88 (80.7%)	0,015
	Situation awareness	652 (65.6%)	87 (79.8%)	0,003
	Teamwork	739 (74.0%)	93 (85.3%)	0,010
	Managing stress	457 (45.8%)	77 (70.6%)	< 0,001
	Coping with fatigue	226 (22.6%)	48 (44.0%)	< 0,001
Assessment of NTSs	Decision-making	529 (53.0%)	78 (71.6%)	< 0,001
	Leadership	508 (50.9%)	74 (67.9%)	< 0,001
	Communication	539 (54.0%)	76 (69.7%)	0,002
	Situation awareness	521 (52.2%)	74 (67,9%)	0,002
	Teamwork	563 (56.4%)	81 (74.3%)	< 0,001
	Managing stress	398 (39.9%)	66 (60.6%)	< 0,001
	Coping with fatigue	199 (19.9%)	46 (42.2%)	< 0,001

**Fig. 3:**
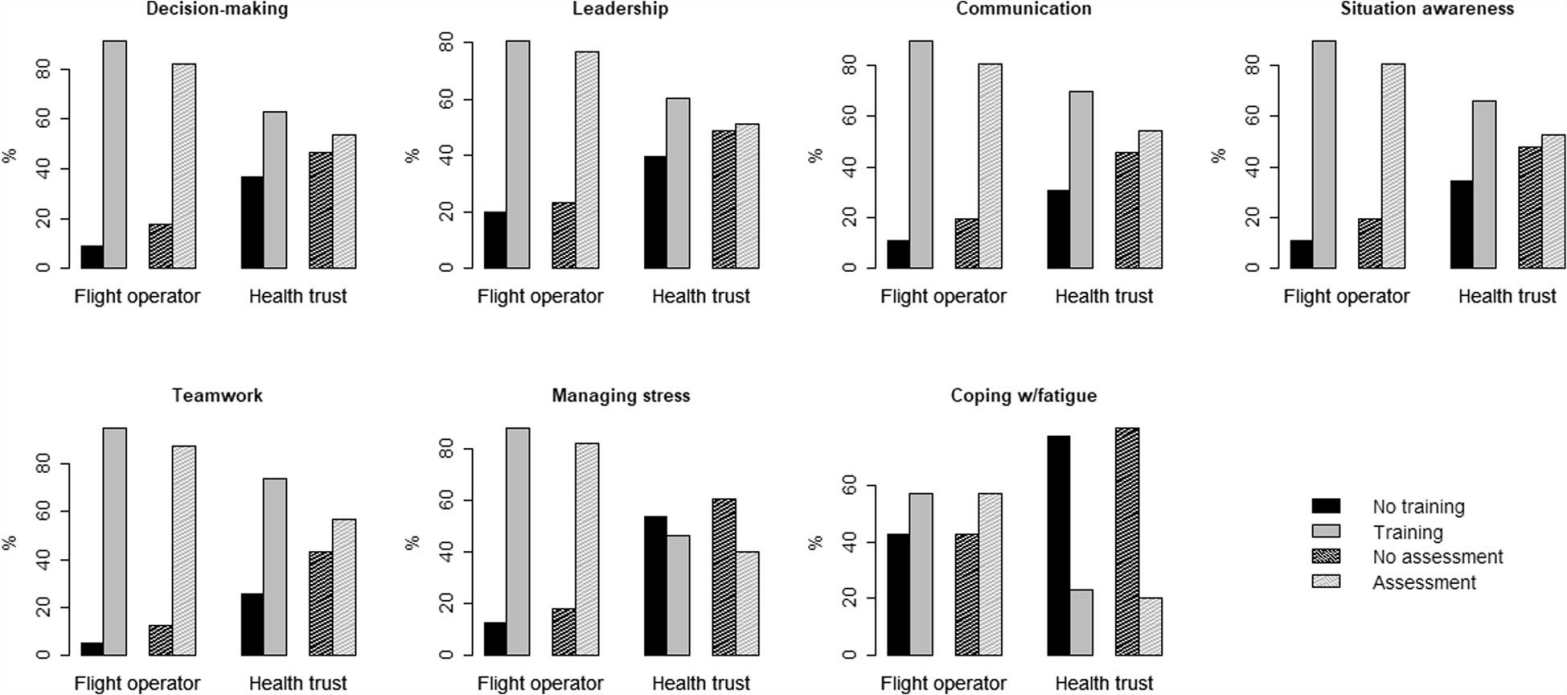
Simulation-based training and assessment (dashed filling) of the seven generic NTSs for employees of the flight operator and health trust. All answers from each of the EMSs are dichotomized into no training/assessment or some training/assessment. Proportions of individuals are the relative frequencies (in %) within each group

The frequency of training in and assessment of the seven NTSs among employees of the flight operator (pilots and HCMs) is statistically significantly greater than for employees of the health trust (Fig. 3 and Table 2).

**Table 2:** Numbers (frequencies) of flight operator and health trust employees undertaking some training in and assessment of the seven NTSs during 2015; Fisher’s exact test proving statistically significant differences

Question category	NTS category	Flight operator employee (*n* = 56)	Health trust employee (*n* = 1051)	*p*-value
	Decision-making	51 (91.1%)	663 (63.1%)	< 0,001
Simulation-based training of NTSs	Leadership	45 (80.4%)	634 (60.3%)	0,003
	Communication	50 (89.3%)	731 (69.6%)	< 0,001
	Situation awareness	50 (89.3%)	692 (65.8%)	< 0,001
	Teamwork	53 (94.6%)	779 (74.1%)	< 0,001
	Managing stress	49 (87.5%)	485 (46.1%)	< 0,001
	Coping with fatigue	32 (57.1%)	242 (23.0%)	< 0,001
Assessment of NTSs	Decision-making	46 (82.1%)	561 (53.4%)	< 0,001
	Leadership	43 (76.8%)	539 (51.3%)	< 0,001
	Communication	45 (80.4%)	570 (54.2%)	< 0,001
	Situation awareness	45 (80.4%)	550 (52.3%)	< 0,001
	Teamwork	49 (87.5%)	592 (56.6%)	< 0,001
	Managing stress	46 (82.1%)	418 (39.8%)	< 0,001
	Coping with fatigue	32 (57.1%)	213 (20.3%)	< 0,001

Compared with the other employees of the health trust (paramedics, EMTs, EMT apprentices, EMT nurses and nurses), physicians appear to undergo training and assessment more frequently. Except for simulation-based training compared with EMT apprentices, these apparent differences are not statistically significant (Table 3).

**Table 3:** ORs with *p*-values, for health trust employees having undergone simulation-based training in and assessment of seven generic NTSs during 2015, compared with the group of physicians (*n* = 53); *p*-values are calculated from logistic regressions

Question category	NTS category	EMT (*n* = 541)		Nurse EMT (*n* = 146)		Nurse (*n* = 37)		Paramedic (*n* = 250)		EMT apprentice (*n* = 24)	
		OR	*p*-value	OR	*p*-value	OR	*p*-value	OR	*p*-value	OR	*p*-value
Simulation- based training of NTSs	Decision-making	0.59	0.107	0.55	0.087	0.75	0.536	0.67	0.242	0.30	0.021
	Leadership	0.77	0.397	0.64	0.183	0.44	0.060	1.09	0.782	0.17	0.001
	Communication	0.88	0.699	0.76	0.430	1.43	0.476	1.06	0.870	0.40	0.068
	Situation awareness	0.88	0.688	0.74	0.376	0.90	0.821	0.82	0.556	0.37	0.048
	Teamwork	0.94	0.853	0.89	0.757	1.18	0.749	0.99	0.978	0.38	0.065
	Managing stress	0.76	0.344	0.74	0.342	1.05	0.909	0.74	0.313	0.54	0.215
	Coping w/fatigue	0.80	0.478	0.62	0.191	0.54	0.231	0.54	0.066	0.21	0.050
Assessment of NTSs	Decision-making	0.73	0.285	0.73	0.339	0.77	0.550	0.80	0.458	0.47	0.130
	Leadership	0.72	0.255	0.69	0.253	0.60	0.242	0.89	0.700	0.36	0.044
	Communication	0.86	0.616	0.77	0.421	0.75	0.503	0.85	0.587	0.60	0.303
	Situation awareness	0.92	0.777	0.87	0.676	0.87	0.753	0.93	0.799	0.59	0.291
	Teamwork	0.88	0.666	0.87	0.656	0.69	0.396	0.82	0.524	0.66	0.395
	Managing stress	1.13	0.648	1.09	0.799	1.40	0.437	0.99	0.985	0.99	0.984
	Coping w/fatigue	0.85	0.632	0.63	0.224	0.65	0.410	0.42	0.018	0.73	0.600

For all the groups and EMSs included in the study, there is a clear tendency for a lower frequency of assessment across all seven generic NTSs, in comparison with the frequency of training (Figs. 2–3 and Tables 1-2).

**Fig. 4:**
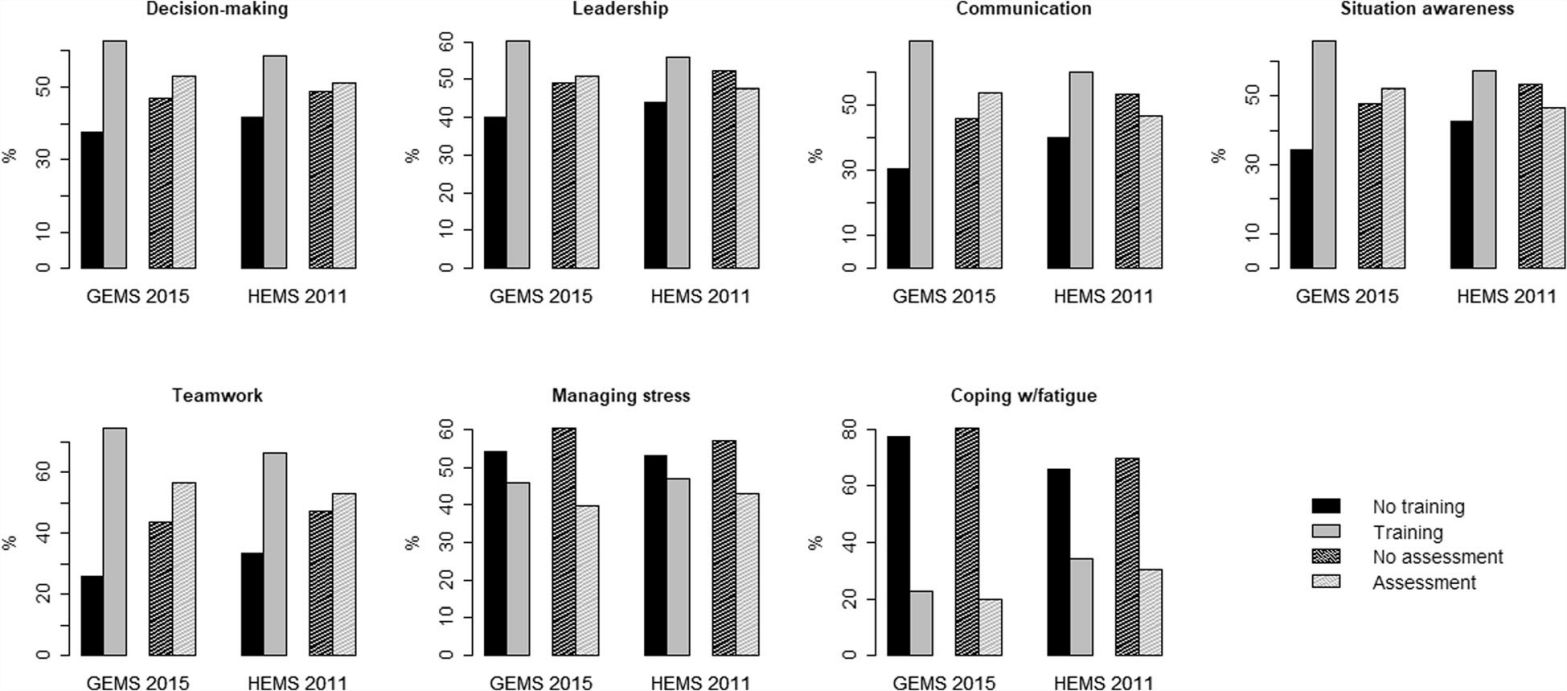
Simulation-based training and assessment (dashed filling) of the seven generic NTSs within the GEMS 2015 and HEMS in 2011. All answers from each of the EMSs are dichotomized into no training/assessment or some training/assessment. Proportions of individuals are the relative frequencies (in %) within each EMS

The frequency of simulation-based training in and assessment of NTSs observed in the HEMS during 2011 [34] is statistically insignificantly different from the frequency observed in the GEMS during 2015 (Fig. 4 and Table 4), except for communication and coping with fatigue. The tendency is that GEMS underwent both training and assessment more frequently during 2015 than HEMS did in 2011.

**Table 4:** Numbers (frequencies) of GEMS employees during 2015 and HEMS employees during 2011 [34] undertaking some training in and assessment of the seven NTSs; Fisher’s exact test proving statistically significant differences

Question category	NTS category	GEMS 2015 (*n* = 998)	HEMS 2011 (*n* = 155)	*p*-value
	Decision-making	624 (62.5%)	87/149 (58.4%)	0,366
Simulation-based training of NTSs	Leadership	599 (60.0%)	84/150 (56.0%)	0,373
	Communication	693 (69.4%)	90/150 (60.0%)	0,024
	Situation awareness	655 (65.6%)	86/159 (57.3%)	0,054
	Teamwork	739 (74.0%)	99/149 (66.4%)	0,060
	Managing stress	457 (45.8%)	80/151 (53.0%)	0,793
	Coping with fatigue	226 (22.6%)	50/146 (34.2%)	0,004
Assessment of NTSs	Decision-making	529 (53.0%)	76/149 (51.0%)	0,661
	Leadership	508 (50.9%)	71/149 (47.7%)	0,483
	Communication	539 (54.0%)	69/148 (46.6%)	0,095
	Situation awareness	521 (52.2%)	69/148 (46.6%)	0,218
	Teamwork	563 (56.4%)	79/149 (53.0%)	0,479
	Managing stress	398 (39.9%)	64/149 (43.0%)	0,475
	Coping with fatigue	199 (19.9%)	44/146 (30.1%)	0,007

## Discussion

The assumption that training and assessment increases NTSs is reasonable based on documented experience [28, 29, 37, 38] and relevant literature [6, 11, 24, 25, 30]. However, direct evidence of improved outcomes or reduced amount of errors as results of training in and assessment of NTSs is sparse in the prehospital domain. The results of this study must be evaluated in respect to the lack of such evidence. The results are also hampered by the low response rate, which makes it ambitious to make strong conclusions. Nevertheless, the tendencies of frequently more training in and assessment of NTSs in HEMS than for GEMS, and that simulation-based training appears to be more frequent than assessment for both the EMSs, are unambiguous.

### Comparison of HEMS and GEMS

The physical environment and task-related differences between HEMS and GEMS may demand different levels of training in NTSs. However, the fact that both prehospital services perform safety-critical operations with a low tolerance of error implies a need for training [11]. One appealing reason for the observed variation in training is the safety culture. NTSs and CRM have been essential features in preventing errors in the aviation industry for a long time [11]. The acknowledgement of human limitations has promoted the need to invest resources in training in NTSs [39]. There is a lack of such strong traditions in GEMS [6], when compared with HEMS, and this may be an explanation of the observed results. Some of the aviation-related tasks performed in HEMS are claimed to be more procedure-based, thus simplifying the simulations and assessments [34]. Training on the base may also be easier to conduct for HEMS than for GEMS, due to the dynamics of the working environment [34].

#### Lack of assessment

In both HEMS and GEMS, our findings show a tendency of less assessment of NTSs than simulation-based training. Just as the effect of simulation-based training should not be underestimated [11], neither should the effect of assessment. Incorrect behaviour, which is not detected, induces the likelihood of errors that could have been prevented [29]. Many of the tasks executed in the EMS are routines, which need to be corrected if they are wrong. Frameworks and tools exist to assess the NTSs in medical teams [37, 40]. However, none are custom-made for the prehospital environment [6]. Proper training without any feedback from qualified personnel can limit the value and learning [41]. In addition to maximize training outcomes, systematic assessment of the NTSs (i.e. debriefing) may detect CRM issues and improve the simulation-based training [42].

#### The greater and lesser focus in the Norwegian HEMS and GEMS

Within both EMSs, the frequency of training in and assessment of teamwork appears to be greater than that of the other NTSs. Strong teamwork is considered fundamental to patient safety and thus, not surprisingly, a major focus, independent of profession and EMS [43]. Each member of an EMS team needs to be aware of his/her own and the other team members’ roles and tasks, to ensure effective and safe patient care, as the safety in the EMS domain relies on mutual understanding among the team members [25].

Our data indicate that less training in and assessment of coping with fatigue, compared to the other NTSs, is present in both HEMS and GEMS, which was also observed in HEMS during 2011 [34]. The previous study [34], reflected on coping with fatigue as not being an explicit NTS category per se but rather an influencer affecting the other NTSs. This study supports such an argument. Fatigue, which is common in the EMS environment [44], ultimately threatens the other NTSs [45], such as teamwork [40]. Therefore, the post-assessment of simulations that comprise fatigue is important, to exploit the full potential of the simulations.

### Comparison of the health trust and flight operator

Compared with flight operator employees, i.e. pilots and HCMs, our data indicate that personnel in the health trust undergo training and assessment significantly less frequently. A possible explanation is the difference in safety culture, as already mentioned. However, a strong safety culture is not a persistent quality that a group achieves automatically. It is a result of focus, resources and commitment over time. In the Norwegian HEMS, significant amounts of resources have been allocated in recent years to enhance training and NTSs [46]. Daily clinical duties in the health trust appear to be less suitable for simulation-based training [34], as it will be too time-consuming and costly, hampering the amount of CRM interventions. However, in situ simulation-based training during on-call hours in the Norwegian HEMS has proven to be feasible [47]. Future research may address the possibility for the health trust to learn from HEMS and adopt similar interventions in their daily duties.

#### Professional requirements

The greater frequency of training in and assessment of NTSs observed among flight operator employees, compared to health trust employees, is no surprise. This tendency was also identified in the study from 2011 [34]. Major disincentives of performing simulation-based training are associated with time-consumption, interruption of daily duties and increased overall expenses [48]. Standards that specify requirements of training may inhibit these natural disincentives, and promote participation in training among the EMS personnel.

In the Norwegian HEMS, standards related to the competency of each profession (i.e. pilot, HCM [49], anaesthesiologist [50]) are established. Within these standards, regular interdisciplinary training is emphasised to achieve high quality health care [49]. The standards are perceived as guiding norms [2], that intend to ensure adequate skills among the personnel in the Norwegian HEMS. In the Norwegian GEMS, basic requirements related to education and skills of each profession are also established [2], but without any further specifications on training, frequency of training, and development of the necessary prehospital skills of GEMS personnel. We call for more research related to establishing such standards in GEMS, which can be motivated by the ones in HEMS.

### Comparison of health trust employees

Among the health trust employees, physicians appear to undergo the greatest amount of training in and assessment of NTSs. The intuitive explanation is the close relationship these physicians have to the aviation safety culture through their experience from HEMS. Although the anaesthesiologists are not obligated to participate in the training conducted in HEMS, which is mandatory for the pilots and HCMs, it is strongly recommended. Opportunities to participate in training and being a member of a culture with a focus on NTSs may have induced an awareness of training and assessment among the physicians.

Equal backgrounds and similarities in responsibility may hamper the development and learning within the team. Diversity in a team can raise awareness of differences and has a positive effect on learning [51, 52]. For GEMS, it can be beneficial to establish a closer relationship with HEMS, with respect to training, in order to promote awareness of their own capabilities and NTSs.

There is an ongoing debate in the Norwegian prehospital domain regarding EMT apprentices conducting on-the-job practice (following the first draft of the Norwegian prehospital emergency medicine regulation [3]), as they are still pursuing their licence. If NTSs are considered to be one of the vaguely stated “required qualities” in the Norwegian prehospital emergency medicine regulation for EMS personnel [3], we may question whether EMT apprentices are eligible to participate in emergency missions. The frequency of training in and assessment of NTSs for EMT apprentices indicates a substantial lack of focus on NTSs and CRM interventions in their education. Our data imply a potential for improvement of simulation-based training in and assessment of NTSs among the EMT apprentices, which the educational programmes in Norway need to be aware of.

### An opportunity for GEMS

There has been an increasing focus on the importance of NTSs and simulations to ensure safety within the Norwegian HEMS over recent years. Specific initiatives have been launched to increase the frequency of training in and assessment of NTSs. In 2011, the Norwegian air ambulance foundation established Camp Torpomoen [53], which is an intensive training programme, in which pilots, HCMs and anaesthesiologists practise together on rare and challenging tasks in safe environments. Another NTS-related initiative in HEMS is the Fatigue Risk Management Programme, initiated in 2013, with the purpose of documenting the physical impact of the working environment and sleep deprivation.

Interestingly, our data imply that the frequency of simulation-based training and assessment observed in HEMS during 2011 [34] is statistically insignificantly different from the frequency observed in GEMS during 2015 (Fig. 4 and Table 4), except for the cases of communication and coping with fatigue. The tendency is that GEMS underwent both training and assessment more frequently during 2015 than HEMS did in 2011. Before the great NTSs-offensive in the Norwegian HEMS, the data indicate that there was no particular association between working in either HEMS or GEMS and the frequency of simulation-based training in and assessment of NTSs. Based on this study, there are now reasons to think otherwise. The potential for learning across the EMSs appears to be present, and it may be a great opportunity for GEMS to gain experience from HEMS. However, the practice applied in HEMS should be adjusted to better fit the GEMS environment, as simulations need to be specifically designed to incorporate significant differences across the two domains [6, 20].

### Limitations and strengths of the study

The response rate among HEMS employees was substantially lower than for the previous study in 2011. This calls into question the representativeness of the 2015 HEMS population. Only one fourth of the GEMS population was included in this study. It is ambitious not to consider non-respondents bias having an impact on the results. However, the observed trends are consistent and unambiguous.

The number of respondents who answered “Other” about their profession, due to having achieved/executing more than one, was significant (*n* = 63). Respondents with “Other” as a profession were categorized manually based on specifications in a free text answer. We had also omitted “EMT apprentice” in the predefined professions in the questionnaire. 24 respondents wrote “EMT apprentice” (“Ambulanselærling”) in a free text field, which we manually categorized as a unique group.

Comparing professions in HEMS and GEMS involves a challenge in sample sizes. It is reasonable to believe that the smaller sub-populations, e.g. pilots, have affected the significance of some results. On the other hand, non-parametric statistical tests, such as the Fisher’s exact test and odds ratio, are resilient to different sample size, strengthening our results.

Dichotomizing the question items into no training/assessment and some training/assessment, reduced the possibility of over- or underreporting, due to respondents not remembering how many times they actually underwent training or assessment.

The questionnaire was tested on a group of seven prehospital healthcare workers to ensure correct terminology. We assumed that all the respondents understood the questions, as no additional explanations and definitions were provided. HEMS employees are more familiar with CRM training and may have better understood what the questions were referring to. The possibility that respondents did not report truthfully is also present.

A weakness of the study, which was not the intention, is the possibility of making strong conclusions regarding the quality of NTSs among the EMSs in Norway. Ultimately, it is the quality, and not the frequency of training and assessment, of the NTSs that the individuals possess which is important.

## Conclusion

The study may help to inform future practice of simulation-based training and assessment in the Norwegian prehospital EMSs, particularly in GEMS. The observed difference in frequency of simulation-based training in and assessment of NTSs in HEMS, compared to GEMS, implies a potential for learning across domains. In both EMSs, the frequency of assessment was significantly lower than for simulation-based training. Special emphasis on how to increase the frequency of assessment is called for to increase the benefits of simulation-based training in NTSs.

## Abbreviations

ALS: Advanced Life Support
CRM: Crew Resource Management
ECTS: European Credit Transfer and Accumulation System
EMS: Emergency Medical Service
EMT: Emergency Medical Technician
GEMS: Ground Emergency Medical Service
HCM: HEMS Crew Member
HEMS: Helicopter Emergency Medical Service
HSOPSC: Hospital Survey on Patient Safety Culture
NTS: Non-Technical Skill
OR: Odds Ratio
SAR: Search and Rescue
